# *p*-Hydroxybenzoic Acid Ameliorates Colitis by Improving the Mucosal Barrier in a Gut Microbiota-Dependent Manner

**DOI:** 10.3390/nu14245383

**Published:** 2022-12-18

**Authors:** Xue Han, Miaomiao Li, Lijun Sun, Xinjuan Liu, Yue Yin, Jianyu Hao, Weizhen Zhang

**Affiliations:** 1 Department of Physiology and Pathophysiology, Peking University Health Science Center, Beijing 100191, China; 2 Department of Gastroenterology, Beijing Chao-Yang Hospital, Capital Medical University, Beijing 100020, China

**Keywords:** colitis, goblet cells, gut microbiota, mucosal barrier, *p*-hydroxybenzoic acid

## Abstract

Inflammatory bowel disease (IBD) is a chronic intestinal inflammatory disease characterized by intestinal inflammatory cell infiltration and intestinal mucosal damage. The mechanism by which diet contributes to the pathogenesis of IBD remains largely unknown. In this study, we explored the therapeutic effect of *p*-hydroxybenzoic acid (HA), a phenolic acid mainly derived from dietary polyphenols in the gut, on DSS-induced colitis. HA intervention effectively relieved the dextran sulfate sodium salt (DSS)-induced colitis, reduced inflammation, and enhanced mucosal barrier function, as evidenced by an increment of goblet cell numbers and MUC2. These effects were largely dependent on the gut microbiota (GM), as antibiotics treatment substantially attenuated the improvement of colitis by HA. On the other hand, transplantation of GM from colitis mice treated with HA significantly reduced the colitis induced by DSS. Our study demonstrates that HA ameliorates DSS-induced colitis by improving the mucosal barrier in a GM-dependent manner. This study provides new dietary choices for the prevention and treatment of IBD.

## 1. Introduction

Inflammatory bowel disease (IBD), including Ulcerative Colitis and Crohn’s Disease, is a chronic inflammatory disease of the gastrointestinal tract. This disease is mainly characterized by weight loss, bloody diarrhea, abdominal pain, and mucus discharge [1]. IBD has been considered a disease in the Western world. However, research over the past decade has indicated an emergence in the Eastern world, especially in Asia. It is estimated that Asia alone might have more cases than the Western world before 2050 [2,3]. Despite its nature as a global epidemic, the current treatment of IBD is limited due to a lack of understanding of its pathogenesis. 

IBD is caused by a combination of genetic, environmental, and immunologic factors and the intestinal barrier [4,5]. Current clinical treatment of IBD mainly focuses on alleviating the symptoms of IBD, especially on reducing intestinal inflammation by suppressing the host’s immune response. Diet is one of the crucial environmental factors in the prevention and management of IBD [6]. Compared to the Mediterranean diet, which is characterized by a high intake of fruits and vegetables, whole grains, and seafood, the Western diet increases the risk of IBD, which is characterized by a high intake of red meat and processed food, saturated fat, and refined sugar. This likely occurs by directly triggering a pro-inflammatory environment [7]. Meanwhile, exclusive enteral nutrition, or its modification, has been widely used in the daily management of IBD [8]. Thus, dietary interventions may hold promise in the prevention and management of IBD. 

Dietary polyphenols are natural antioxidants. Many of them, including phenolic acids, flavones, and anthocyanins, have a variety of physiological functions. *P*-Hydroxybenzoic acid (HA), a type of phenolic acid, is rich in nuts, seeds, leafy vegetables, and tea. The daily intake of HA from this food for human beings is estimated to be 25–100 mg [9]. HA can also be generated as one of the metabolites of dietary polyphenols in vivo [10,11]. Previous studies have shown the antifungal, antimutagenic, antioxidant, and anti-obesity effects of HA [12,13,14]. Studies by Xiaotian Xu et al. have demonstrated that HA is an active substance capable of inhibiting inflammatory responses and improving intestinal mucosal damage via the activation of Erβ signaling in dextran sulfate sodium salt (DSS)-induced colitis [15]. Whether HA has a new mechanism for relieving colitis remains to be explored. In this study, we reported the beneficial effect and new mechanism of HA on DSS-induced colitis. This effect occurred via restoration of the intestinal mucosal barrier in a manner dependent on the gut microbiota (GM), especially *Akkermansia muciniphila* (*A. muciniphila*). 

## 2. Materials and Methods

### 2.1. Experimental Animals and Dosage Information

Eight-week-old male C57BL/6J mice were purchased from Vital River Laboratory Animal Technology (Beijing, China). Mice were maintained in a 12/12 h light-dark cycle with free access to water and a standard chow diet (Beijing Keao Xieli Feed Co., Ltd., Beijing, China). The composition of the standard chow diet is shown in Supplementary Table S1. Colitis was induced by administering 2.5% DSS (molecular weight 36,000–50,000 kDa, MP Biomedicals, Santa Ana, CA, USA) dissolved in drinking water. 

The following three animal experiments were conducted in this study:

(1) To examine the therapeutic effect of HA, mice were divided into the following three groups: the Control group, the DSS group (2.5% DSS dissolved in drinking water), and the DSS + HA group (HA purchased from TCI (H0207, Shanghai, China). HA was administrated at a dose of 100 mg/kg body weight by oral gavage for 7 days, then DSS and HA were administered for another 7 days.
(2) To determine whether the effect of HA was dependent on GM, mice were pretreated with antibiotic cocktails (1 g/L ampicillin, 1 g/L neomycin, 0.5 g/L meropenem, and 0.5 g/L vancomycin dissolved in drinking water) for 7 days. Animals were subsequently fed with drinking water containing antibiotic cocktails and 2.5% DSS with or without gavage HA for 6 days.
(3) For the fecal microbiota transplantation (FMT) experiment, mice were divided into the following four groups: control (Ctr, PBS gavage), DSS group (2.5% DSS dissolved in drinking water with PBS gavage), DSS + NC FMT (2.5% DSS dissolved in drinking water and FMT of DSS group feces) and DSS + HA FMT (2.5% DSS dissolved in drinking water and FMT of DSS + HA group feces). The fecal microbial solution was prepared as the supernatant after the natural settlement of 200 mg of fresh feces mashed in 5 mL sterile PBS. A total of 200 μL of fecal microbiota was transplanted to each mouse every other day. After three transplantations, 2.5% DSS was administered for 7 days to induce colitis.

The disease activity index (DAI) was evaluated by combining the parameters of weight loss percentage, fecal sparsity, and rectal bleeding before sacrifice [16]. At the end of the experiment, mice were sacrificed by performing intraperitoneal injection of pentobarbital sodium (60 mg/kg) and cardiac exsanguination. Serum, cecal contents, and colon samples were collected.

### 2.2. Histological and Immunohistochemical Analysis

Colon samples were fixed in 4% paraformaldehyde and embedded in paraffin. Multiple sections were prepared and stained with hematoxylin and eosin for morphological examination. Images were taken using the Nano Zoomer microscope (Hamamatsu Photonics, Hamamatsu, Japan). The pathological score was evaluated based on an established scoring system (0–5 score) including loss of the epithelial surface, destruction of the crypt, and infiltration of immunocytes.

Immunohistochemical analysis of MUC2 (anti-MUC2, GB11344, Servicebio, Wuhan, China) in the colon was carried out according to a standard protocol. The MUC2-positive area under each field was calculated and ten fields per mouse were counted.

### 2.3. Quantitative Real-Time PCR

The total RNA was extracted with TRIGene Reagent (GenStar, Beijing, China). Reverse transcription was performed using the HiScript II 1st Strand cDNA Synthesis Kit (R211, Vazyme, Nanjing, China). Relative gene expression was measured with the Taq Pro Universal SYBR qPCR Master Mix (P211, Vazyme, Nanjing, China) using the Aria Real-Time PCR System (Agilent Technologies, Santa Clara, CA, USA). Data were normalized to *β-*actin and analyzed using the ΔΔCt method. The primer sequences are shown in Supplementary Table S2.

### 2.4. Flow Cytometry

Colons were collected and analyzed by performing flow cytometry for the proportion of T cells. Briefly, the colon was dissected longitudinally and washed in PBS to remove its contents. The tissue was incubated in an RPMI 1640 medium (BBI, Shanghai, China) containing 5 mmol/L DTT (Beyotime Biotechnology, Shanghai, China), 1 mmol/L EDTA (TGREGA, Beijing, China), and 5% HyClone™ fetal bovine serum (Cytiva, Marlborough, MA, USA) for 40 min at 37 °C. After washing with medium, the colon was minced in a RPMI 1640 medium containing 1 mg/mL collagenase I and 0.1 mg/mL Dnase I and incubated for 30 min at 37 °C. After termination of digestion, the solution was filtered through a 70 μm cell strainer. After centrifugation at 500× *g* for 10 min, single cells were obtained in the pellet. 

After induction in the medium (RPM1640 supplemented with Cell Stimulation Mix (00-4970-93, Invitrogen, Carlsbad, CA, USA)) for 4 h in a 37 °C incubator containing 5% CO_2_, cells were stained for 15 min at room temperature using the APC/cy 7 Fixable Viability Kit (423106, Biolegend, San Diego, CA, USA) for live cell staining. Next, cells were stained with anti-mouse CD16/32 (101320, Biolegend, San Diego, CA, USA), PE/cy5 anti-mouse CD45 (103109, Biolegend, San Diego, CA, USA), and FITC anti-mouse CD4 (100509, Biolegend, San Diego, CA, USA) for 40 min at 4 °C. Then, cells were permeabilized with Fix/Perm buffer (562574, BD Biosciences, Franklin Lakes, NJ, USA) for 40 min at 4 °C and stained with PE anti-mouse FOXP3 (126403, Biolegend, San Diego, CA, USA) and PE/cy 7 anti-mouse IL17A (506921, Biolegend, San Diego, CA, USA) for 50 min at 4 °C. Samples were analyzed using a Flow Cytometer (BD Biosciences, Franklin Lakes, NJ, USA). Results were analyzed with FlowJo software V10 (BD Biosciences, Ashland, OR, USA). Treg cells were identified as CD45^+^ CD4^+^ FOXP3^+^. Th17 cells were identified as CD45^+^ CD4^+^ IL17A^+^.

### 2.5. Analysis of Inflammatory Cytokines

Serum levels of cytokines including IL4 (KE10010), IL6 (KE10007), IL10 (KE10008), IL17A (KE10020), and TNFa (KE10002), were measured with the ELISA kit (Proteintech, Wuhan, China) according to the manufacturer’s instructions.

### 2.6. PAS-AB Staining and Analysis

Colon PAS-AB staining was performed according to the instructions of the AB-PAS Stain Kit (G1285, Solarbio Science & Technology, Beijing, China). Images were taken using the Nano Zoomer microscope (Hamamatsu Photonics, Hamamatsu, Japan). The number of goblet cells was counted in 80–100 crypts per mouse.

### 2.7. 16S rRNA Gene Sequencing and Analysis

DNA isolation, PCR amplification, and sequencing were performed as described previously [17]. Briefly, after the total genomic DNA of cecal contents was extracted using the CTAB/SDS method, and 16S /18S rRNA genes were amplified using the specific primer with the barcode. All PCR reactions were carried out using the Phusion^®^ High-Fidelity PCR Master Mix (New England Biolabs, Ipswich, MA, USA). PCR products were purified with the GeneJET Gel Extraction Kit (Thermo Scientific, Carlsbad, CA, USA). Sequencing libraries were generated using the Illumina TruSeq DNA PCR-Free Library Preparation Kit (Illumina, San Diego, CA USA) following the manufacturer’s recommendations, and index codes were added. The library quality was assessed with the Qubit@ 2.0 Fluorometer (Thermo Scientific, Carlsbad, CA, USA) and Agilent Bioanalyzer 2100 system (Agilent, Santa Clara, CA, USA). Finally, the library was sequenced on an Illumina Nova Seq platform and 250 bp paired-end reads were generated. Sequences with >97% similarity were assigned to the same OTUs. Sequences were analyzed using the QIIME2 software package. The accession number for the data reported in this study is https://doi.org/10.6084/m9.figshare.21251127.v1 (accessed on 30 September 2022).

### 2.8. Statistics

Data were expressed as mean ± SE. Significant differences between the two groups were evaluated with the *t*-test. Significant differences in more than two groups were evaluated with a one-way ANOVA test followed by Duncan’s test. A *p* value of < 0.05 was considered statistically significant. Statistical analyses were performed using SPSS 22.

## 3. Results

### 3.1. HA Ameliorates Colitis Induced by DSS

**Figure 1:**
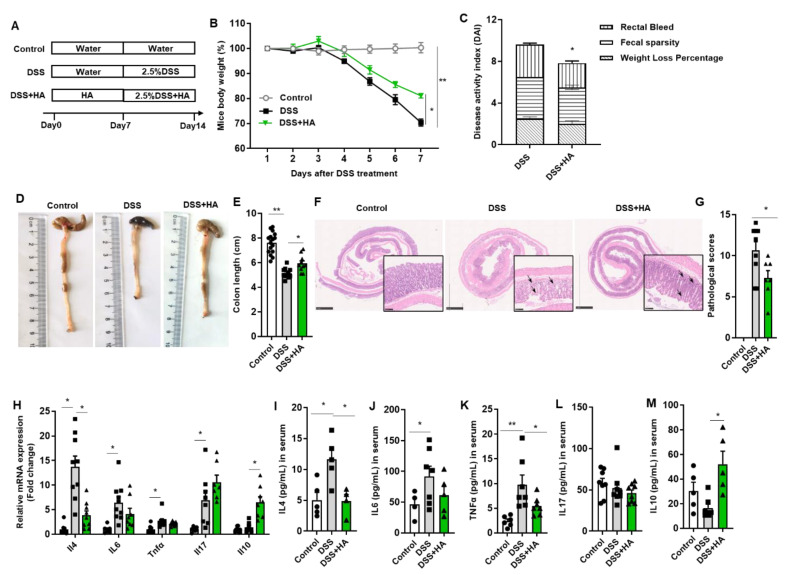
HA ameliorates colitis induced by DSS. (**A**) Experimental design. (**B**) Percentage change in body weight after DSS treatment. (**C**) Disease activity index on day 7. (**D**,**E**) Representative images and length of the colon. (**F**,**G**) Representative image of H&E staining of colon and pathological score. The right lower panels (20×) are images enlarged from the low magnification panels (2.5×). (**H**) Relative mRNA expression of inflammatory factors in the colon. (**I**–**M**) Concentrations of IL4, IL6, TNFa, IL17, and IL10 in serum. Data are expressed as mean ± SE, n = 8–10, * *p* < 0.05, ** *p* < 0.01.

To investigate the effects of HA on DSS-induced colitis, mice were pretreated with HA for 7 days; subsequently, 2.5% DSS plus HA was administered for the next 7 days (Figure 1A). PBS was used as the control. As shown in Figure 1B, HA significantly reduced the body weight loss relevant to animals gavaged with PBS. The DAI score, assessed by rectal bleeding, fecal sparsity, and body weight loss, also markedly decreased with HA (Figure 1C). Further, the reduction in colon length was significantly attenuated (Figure 1D,E). Histologically, HA treatment notably reduced the loss of the epithelial surface, destruction of the crypt, and infiltration of immunocytes induced by DSS (Figure 1F,G). 

Previous studies have indicated that Th17 and Treg cells are critical for the pathogenesis of IBD [18]. We thus examined the effects of HA on Th17 (CD45^+^ CD4^+^ IL17A^+^) and Treg (CD45^+^ CD4^+^ FOXP3^+^) cells in the intestinal lamina. The proportion of Th17 and Treg cells decreased significantly in HA-treated mice compared to vehicle control mice (Figure S1A,B), suggesting an amelioration in the inflammatory response. In line with this, mRNA levels of proinflammatory cytokines including IL4, IL6, and Tnfa in the colon, as well as their serum protein levels, were notably decreased in mice treated with HA (Figure 1H–K). However, HA demonstrated no significant effect on IL17 (Figure 1H,L). On the other hand, HA treatment significantly increased the colonic mRNA levels and the serum concentration of IL-10 (Figure 1H,M), a key anti-inflammatory cytokine.

Taken together, our observation demonstrates that HA ameliorates colitis induced by DSS in mice.

### 3.2. HA Improves Mucosal Barrier 

**Figure 2:**
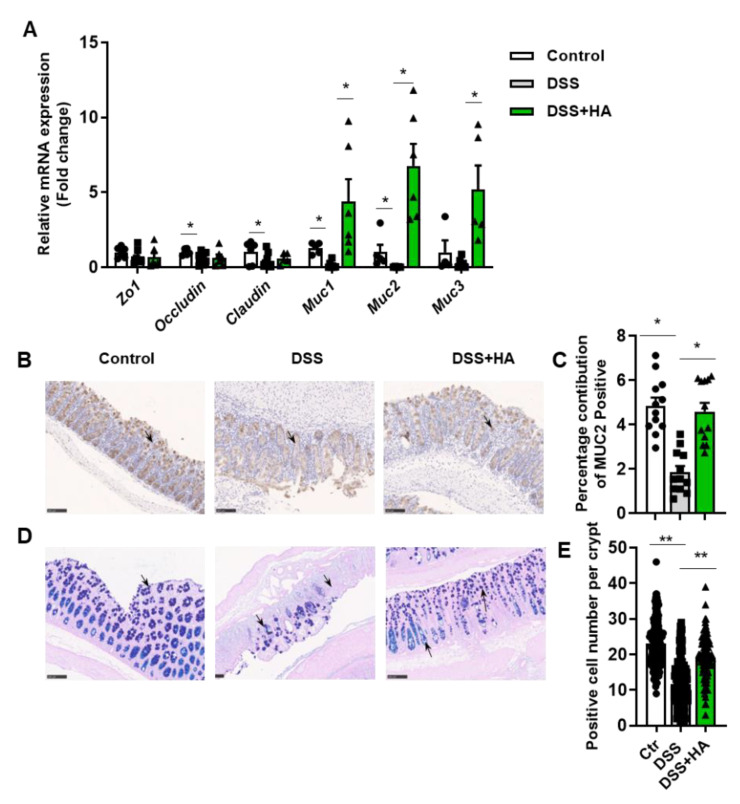
HA improves mucosal barrier. (**A**) Relative mRNA expression of tight junction genes and mucin genes in the colon. (**B**,**C**) Immunohistochemical analysis of MUC2 in the colon (20×). (**D**,**E**) PAS-AB staining of the colon (20×). Data are expressed as mean ± SE, n = 8–10, * *p* < 0.05, ** *p* < 0.01.

A tight junction, composed of claudin, occluding, Zos, other structural proteins, and various connexin molecules, is the most important structure constituting the mucosal mechanical barrier. Compared with the control group, DSS treatment reduced the mRNA expressions of certain tight junction proteins in the colon, including Occludin and Claudin (Figure 2A). Interestingly, HA demonstrated no significant effects on the decrement of tight junction gene mRNA in the colon (Figure 2A). Instead, it completely reversed the reduction in the mRNA expression of mucins (MUCs), including Muc1, Muc2, and Muc3 induced by DSS (Figure 2A). Consistently, MUC2 protein expression in colonic tissue was significantly increased by HA (Figure 2B,C). Further evaluation of goblet cells by performing PAS-AB staining showed that HA significantly attenuated the reduction in goblet cells observed in mice treated with DSS (Figure 2D,E). These results indicate that HA administration improves the mucosal barrier by increasing the number of goblet cells, leading to an increment in MUC, especially MUC2.

### 3.3. Antibiotics Treatment Blunts the Effects of HA

**Figure 3:**
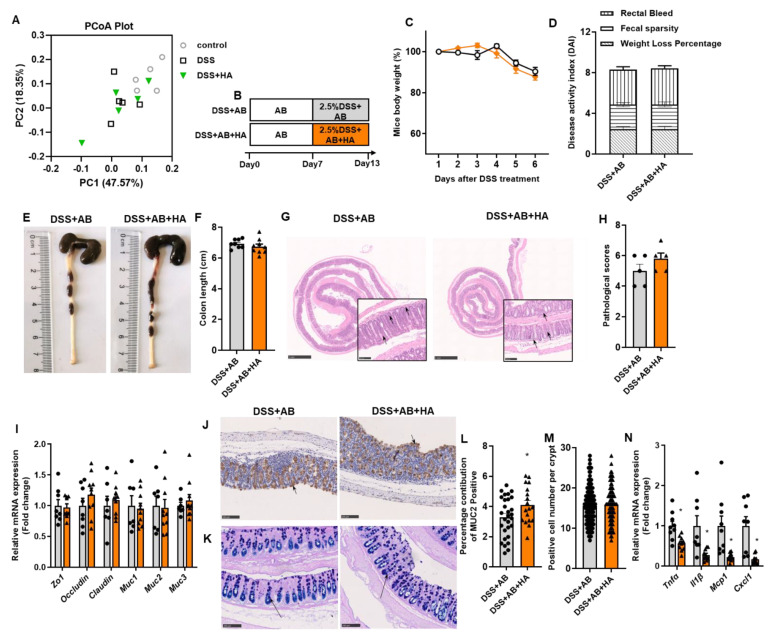
Antibiotics treatment blunts the effects of HA. (**A**) The β-diversity analysis of GM by principal component analysis (PCA) plot of Control, DSS, and DSS + HA (*n* = 4–5). (**B**) Experimental design of antibiotics (AB) treatment. (**C**) Percentage change in body weight after DSS treatment. (**D**) Disease activity index (DAI) on day 7. (**E**,**F**) Representative image and length of the colon. (**G**,**H**) Representative image of H&E staining of colon and pathological score. The small panels (20×) were enlarged from the large panels (2.5×). (**I**) Relative mRNA expression of tight junction genes and mucin genes in the colon. (**J**,**L**) Immunohistochemical analysis of MUC2 in the colon (20×). (**K**,**M**) PAS-AB staining of the colon (20×). (**N**) Relative mRNA expression of inflammatory factors in the colon. Data are expressed as mean ± SE, n = 8–10, * *p* < 0.05.

Gut microbial dysbiosis has been identified as an important pathogenic factor in IBD [19]. HA intervention demonstrated no significant influence on the α-diversity of GM (Figure S2). The weighted principal co-ordinates analysis (PcoA) showed that there was no significant change between the HA-treated group and the DSS group (Anosim test, *p*-value = 0.62) (Figure 3A). We then explored whether the HA-mediated improvement of colitis is dependent on GM by eliminating intestinal bacteria using antibiotic (AB) cocktails. Mice were pretreated with AB for 7 days; they then received AB and 2.5% DSS in drinking water with the gavage of HA or PBS for 6 days (Figure 3B). As shown in Figure 3, elimination of GM blocked the beneficial effect of HA on DSS-induced colitis. There were no significant differences in body weight change, rectal bleeding, and fecal sparsity between the two groups (Figure 3C,D). In addition, the beneficial effect of HA on colon length, H&E staining pathology score, MUC2 mRNA, and protein expressions, as well as mucus-positive colonic goblet cells were substantially blunted by AB (Figure 3E–M). In the presence of AB, the HA supplement still significantly reduced the colonic mRNA levels of inflammatory factors, including Tnfa, Il1β, Mcp1, and Cxcl1 (Figure 3N), suggesting that the beneficial effects of HA may not depend on the amelioration of colonic cytokines.

### 3.4. Fecal Microbiota Transplants (FMT) from Colitis Mice Treated with HA Ameliorate Colitis by Increasing the Abundance of Akkermansia muciniphila 

**Figure 4:**
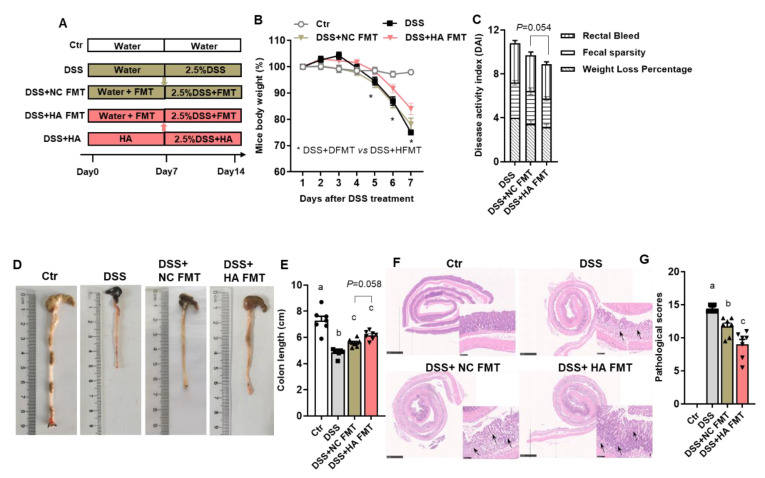
Microbiota Transplants (FMT) from colitis mice treated with HA ameliorate colitis. (**A**) Experimental design. (**B**) Percentage change in body weight after DSS treatment. (**C**) Disease activity index on day 7. (**D**,**E**) Representative images and length of the colon. (**F**,**G**) Representative image of H&E staining of colon and pathological score. The small panels (20×) were enlarged from the large panels (2.5×). Data are expressed as mean ± SE, n = 8–10, and significant differences (*p* < 0.05) are denoted by a,b,c.

To further demonstrate that the relieving effect of HA on colitis is dependent on GM, fecal microbiota transplant (FMT) experiments were performed (Figure 4A). Mice transplanted with FM harvested from colitis animals treated with HA (HA + FMT group) demonstrated a significant increment in body weight as well as a decrement in DAI scores relevant to the control NC + FMT group (Figure 4B,C). In addition, colon length increased markedly in the HA + FMT group (Figure 4D,E). Moreover, the transplantation of FM from colitis mice treated with HA significantly attenuated the loss of epithelial surface, destruction of the crypt, and the infiltration of immunocytes compared to the transplantation of the microbiome from colitis mice treated with control (Figure 4F,G). 

**Figure 5:**
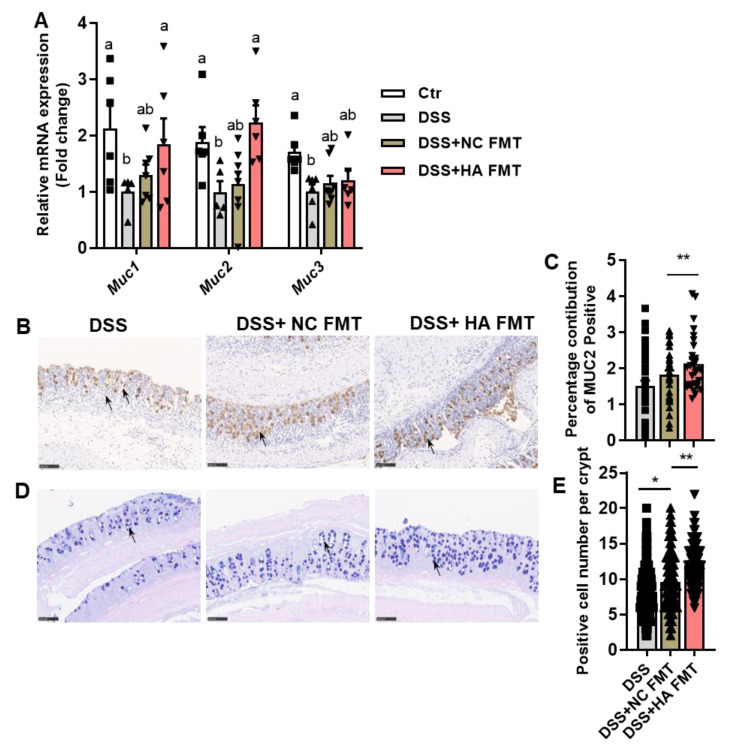
FMT from colitis mice treated with HA improves the mucosal barrier. (**A**) Relative mRNA expression of mucin genes in the colon. (**B**,**C**) Immunohistochemical analysis of MUC2 in the colon (20×). (**D**,**E**) PAS-AB staining of the colon (20×). Data are expressed as mean ± SE, n = 8–10, * *p* < 0.05, ** *p* < 0.01, lowercase letters (a,b) indicate significant differences (*p* < 0.05).

Consistent with the observation that HA administration increased the number of goblet cells and expression of MUC2 in colitis mice (Figure 2), transplantation from HA-treated colitis mice increased the mRNA levels of Muc1 and Muc2 (Figure 5A), as well as the protein expression of MUC2 (Figure 5B,C). The number of mucus-positive goblet cells also increased (Figure 5D,E). This observation indicates that transplantation of FM from colitis mice treated with HA can mimic the direct action of HA administration in the increment of goblet cells and MUC2.

On the other hand, serum IL6 was significantly reduced in the colitis mice receiving FMT from colitis mice treated with HA relevant to the control group (Figure S3A). Other cytokines such as IL4, IL17, TNFa, and IL10 remained unaltered (Figure S3B–E). The Nether change accounted for the proportion of Th17 and Treg cells (Figure S3F,G). 

**Figure 6:**
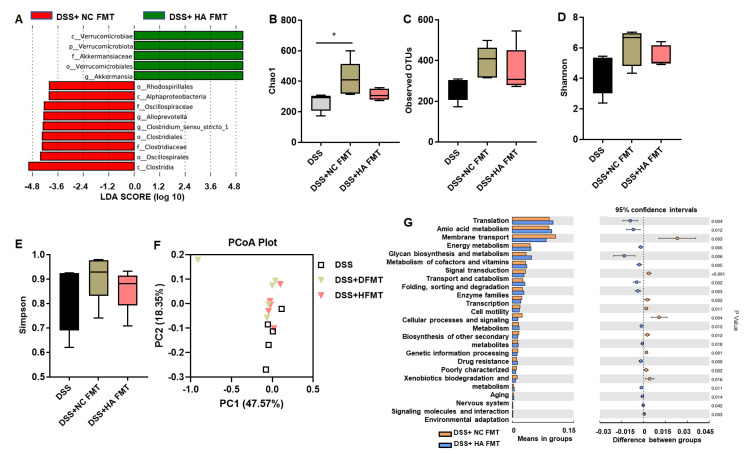
FMT from colitis mice treated with HA increases the abundance of *Akkermansia muciniphila*. (**A**) The LefSe analysis of the DSS + NC FMT group and DSS + HA FMT group. (**B**–**E**) The α-diversity analysis of the gut microbiome at the OTU level, determined by the Chao1, Observed OTUs, Shannon and Simpson index. (**F**) The β-diversity analysis by principal component analysis (PCA) plot. (**G**) The microbial predicted metabolic functional data using Tax4Fun method. Data are expressed as mean ± SE, n = 5, * *p* < 0.05.

We next investigated whether FMT from colitis mice treated with HA relieves DSS-induced colitis via the gut microbiome. The analysis of 16S rRNA of bacteria in cecal contents showed that the abundance of *Akkermansia muciniphila* (*A. muciniphila*) was significantly increased in colitis mice receiving FMT from animals treated with HA (HA + FMT group) compared to the control (NC + FMT group) (Figure 6A). There were no significant differences in the abundance of microbial communities (Figure 6B–E) as well as the microbial construction, as evidenced by the weighted PcoA plot on OUT levels (Figure 6F) between the HA + FMT group and NC + FMT group (Anosim test, *p*-value = 0.27). Microbial function prediction showed that the membrane transport and cell motility of GM in the HA + FMT group were significantly enhanced compared with the NC + FMT group (Figure 6G). These data suggest that FMT from colitis mice treated with HA did not alter the overall abundance of the host microbiota. Instead, it increased the abundance of *A. muciniphila*, suggesting that *A. muciniphila* may contribute to the beneficial effect of HA intervention.

## 4. Discussion

Our study demonstrates that HA intervention is efficient in the amelioration of colitis induced by DSS and that its effect occurred via restoration of the intestinal mucosal barrier in a manner dependent on the GM. This observation extends the beneficial effect of dietary polyphenols in relieving IBD to HA, a phenolic acid mainly derived from dietary polyphenols in the gut. Currently, a variety of animal experimental models are widely used to study the etiology, pathogenesis, and treatment of IBD. DSS-induced colitis is widely used because its symptom and histological changes are very similar to human UC, and the different concentrations, duration, and frequency of administration can lead to two models of acute and chronic colitis. In this study, 8-week-old male C57BL/6 mice were used and 2.5% DSS was diluted into drinking water for 7 days to induce an acute colitis mouse model, which is widely used [16,20,21]. 

Studies by Xiaotian Xu et al. have demonstrated that the oral administration of HA (10–40 mg/kg) dose-dependently attenuated various colitis phenotypes in DSS-treated mice [15]. In our study, the oral administration of HA at a dose of 100 mg/kg body weight was found to be efficient to relieve colitis. In addition to HA, certain phenolic compounds have also been shown to relieve colitis. Ferulic acid or cyclopentenyl ferulate at a dose of 50 mg/kg has been reported to reduce the inflammation in DSS-induced colitis in mice [22]. Studies by Fang et al. have also found the protective effect of syringic acid at the dose of 25 mg/kg body weight [23]. Sinapic acid at a dose ranging from 10 to 100 mg/kg has been demonstrated to alleviate the damage induced by either lipopolysaccharide in Caco-2 cells, or 2,4,6-trinitrobenzene sulfonic acid (TNBS) and DSS in mice [24,25,26]. Chlorogenic acid and gallic acid have been found to have identical efficiencies in the alleviation of interleukin-10 knockout IBD [27,28], as well as colitis induced by TNBS, 1, 2-Dimethylhydrazine (DMH), or DSS [29,30,31]. In addition, the microbial metabolite Urolithin A (20 mg/kg) mitigates DSS-induced acute colitis through its gut barrier protective and anti-inflammatory activities in an aryl hydrocarbon receptor-dependent manner [32,33].

Among the complex mechanisms underlying the etiology of IBD, the gut microbiota is one of the most important factors [5]. DSS caused significant disruption to the GM, which was restored with HA treatment, indicating a possible role of GM in the improvement of colitis by HA. The indispensable role of GM was further verified by the elimination of GM by antibiotics, which blunted the beneficial effect of HA on colitis. Eventually, transplantation of the GM from HA-treated mice transferred the protection to these recipient mice, demonstrating that GM mediated the colitis-improving effect of HA. We found a significant enrichment of *A*. *muciniphila* in mice receiving HA-treated GM. Interestingly, the enrichment of *A*. *muciniphila* has been widely reported after the treatment of various dietary polyphenols, such as polyphenols from blueberry, grape, grape seed, and tea polyphenols [34,35]. Discovered in 2004, *A. muciniphila* has been demonstrated to be a valuable strain of intestinal bacteria critical for a series of chronic diseases such as cancer, diabetes, obesity, premature aging, hypertension, autism, and certainly in IBD. Although it uses mucin as an energy source, it is widely accepted that the *A. muciniphila* bacterium functions to enhance gut homeostasis (for example, by increasing the thickness of the intestinal mucus layer and the integrity of the intestinal barrier); despite this, in certain cases, it may act as an opportunistic microbe [36,37]. 

In line with this concept, our study shows that HA significantly attenuated the impairment of intestinal barriers in mice with DSS-induced colitis. Intestinal barriers include both mechanical and chemical barriers. The mechanical barriers refer to tight junctions between cells, mainly composed of tight junction proteins. In our study, HA intervention did not alter the expression of tight junction proteins. Instead, it increased the number of goblet cells, leading to a subsequent increment in the expression of mucins, especially MUC2 [35,38]. This observation indicates that HA intervention may function to improve the chemical barrier by increasing the number of goblet cells and the subsequent production of mucins. In contrast to our observation, previous studies have shown that phenolic acids relieve IBD mainly by suppressing inflammation rather than through an effect on the mucosal barrier. However, our evaluation of immune responses, including both inflammatory factors and immune cells, indicates that the beneficial effect of HA intervention in colitis may not solely rely on the suppression of inflammation. Although the beneficial effect of HA was greatly attenuated in germ-free mice, it still significantly reduced the mRNA expression of cytokines and chemokines. Furthermore, the transplantation of GM from colitis mice treated with HA demonstrated no effect on the proportions of Th17 and Treg. While these observations indicate a mechanism other than the immune response underlying the therapeutic effect of HA in colitis, it is worth noting that the anti-inflammatory action of HA cannot be excluded. Indeed, HA decreased mucosal mRNA levels and blood concentrations of proinflammatory cytokines such as IL6, and TNFa, while concurrently increasing IL-10, a typical immunosuppressive cytokine. In addition, the proportion of Th17 and Treg cells was significantly reduced following HA supplementation. These results are consistent with the widely recognized concept of the anti-inflammatory effects of polyphenols. Further investigation should focus on the differential effects of distinct polyphenols on the immune response and intestinal barrier.

## 5. Conclusions

In conclusion, our study innovatively demonstrates that HA, a phenolic acid, improves IBD by increasing the number of goblet cells and the thickness of mucins. This likely occurs via a mechanism dependent on the *A. muciniphila*. Our studies thus provide a novel strategy for the intervention of IBD by targeting the HA- *A. muciniphila-*mucins/goblet cells axis. However, the interactions between HA, gut microbiota, and host health need to be further studied [39]. Additionally, more evidence is needed for the subsequent clinical application of HA.

## Supplementary Materials

The following supporting information can be downloaded at: https://www.mdpi.com/article/10.3390/nu14245383/s1, Figure S1: Effects of HA administration on Th17/ Treg cells; Figure S2: HA intervention demonstrates no significant influence on the α-diversity of GM; Figure S3: The effects of FMT from colitis mice treated with HA on inflammatory factors and immune cells; Table S1: The composition of the standard chow diet (per kg), Table S2: Primers sequences.

## Abbreviations

Inflammatory bowel disease: IBD; gut microbiota: GM; *Akkermansia muciniphila*: *A. muciniphila*; *p*-Hydroxybenzoic acid: HA; dextran sulfate sodium salt: DSS; mucin: MUC.
