# Supplementary material for: p-Hydroxybenzoic Acid Ameliorates Colitis by Improving the Mucosal Barrier in a Gut Microbiota-Dependent Manner
Source: Nutrients. 2022 Dec 18;14(24):5383. doi: 10.3390/nu14245383 (PMC9784546; doi:10.3390/nu14245383)
Supplement: Supplementary file 1 [file nutrients-14-05383-s001.zip › nutrients-2049053-supplementary.pdf]

Supplementary Table S1. The composition of the standard chow diet (per kg).

**Vitamin**		**Mineral**	
Vitamin A (IU)	7800	Na (g)	3.1
Vitamin D (IU)	1200	Mg (g)	2.9
Vitamin E (mg)	67	K (g)	7.4
Vitamin K (mg)	5	Cu (mg)	11.4
Vitamin B1 (mg)	10	Fe (mg)	113.7
Vitamin B2 (mg)	15	Mn (mg)	80
Vitamin B6 (mg)	10	Zn (mg)	31.6
Vitamin B12 (mg)	0.02	Se (mg)	0.2
Niacin (mg)	55	I (mg)	0.7
Pantothenic acid (mg)	22	**Proportion of energy supply**	
Biotin (mg)	0.2	Protein	23.07%
Choline (mg)	1250	Fat	11.85%
Folic acid (mg)	6.6	Carbohydrate	65.08%
**Amino acid (g)**		Total	3.4 kcal/g
Methionine + Cystine	5.8		
Lysine	8.9		
Tryptophan	2.1		
Arginine	9.9		
Leucine	14.8		
Isoleucine	7.4		
Threonine	6.6		
Valine	8.9		
Histidine	4.9		
Phenylalanine + tyrosine	14.6		

Supplementary Table S2. Primers sequences.

	Forward Primer	Reverse Primer
β-Actin	GTGACGTTGACATCCGTAAAGA	GCCGGACTCATCGTACTCC
Muc1	GGCATTCGGGCTCCTTTCTT	TGGAGTGGTAGTCGATGCTAAG
Muc2	ATGCCCACCTCCTCAAAGAC	GTAGTTTCCGTTGGAACAGTGAA
Muc3	CCGAGAGCGGAAGTGTGTG	TGTAACTGTGGTTTTGGTCTTCA
Zo-1	GCCGCTAAGAGCACAGCAA	GCCCTCCTTTTAACACATCAGA
Occludin	TGAAAGTCCACCTCCTTACAGA	CCGGATAAAAAGAGTACGCTGG
Claudin	TGCCCCAGTGGAAGATTTACT	CTTTGCGAAACGCAGGACAT
Tnfα	CAGGCGGTGCCTATGTCTC	CGATCACCCCGAAGTTCAGTAG
Il1β	GAAATGCCACCTTTTGACAGTG	TGGATGCTCTCATCAGGACAG
Il10	CTTACTGACTGGCATGAGGATCA	GCAGCTCTAGGAGCATGTGG
Cxcl1	ACTGCACCCAAACCGAAGTC	TGGGGACACCTTTTAGCATCTT
Il17a	TCAGCGTGTCCAAACACTGAG	CGCCAAGGGAGTTAAAGACTT
IL4	GGTCTCAACCCCCAGCTAGT	GCCGATGATCTCTCTCAAGTGAT
IL6	CTGCAAGAGACTTCCATCCAG	AGTGGTATAGACAGGTCTGTTGG
Mcp1	TAAAAACCTGGATCGGAACCAAA	GCATTAGCTTCAGATTTACGGGT

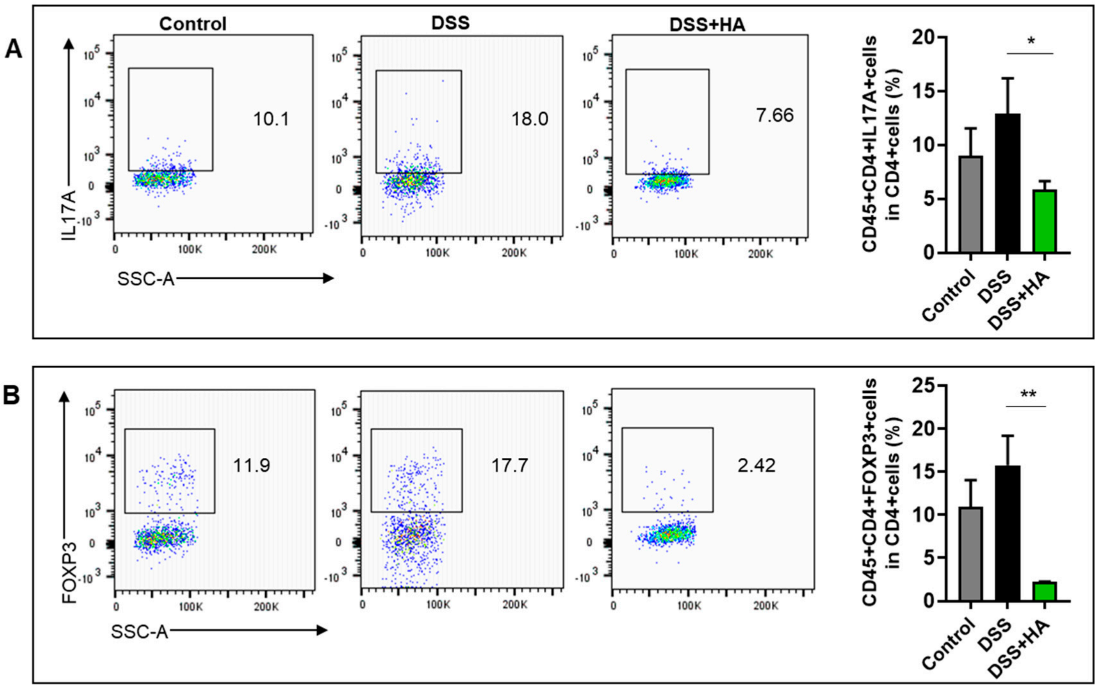

Figure S1. Effects of HA administration on Th17/ Treg cells. (**A** and **B**) The proportion of Th17 (CD45+ CD4+ IL17A+) cells and Treg (CD45+ CD4+FOXP3+) cells. The color of the dots represents the frequency of cell occurrence. Redder represents greater cell density. Data are expressed as mean±SE, n = 4−6; *, $P<0.05$; **,$P<0.01$.

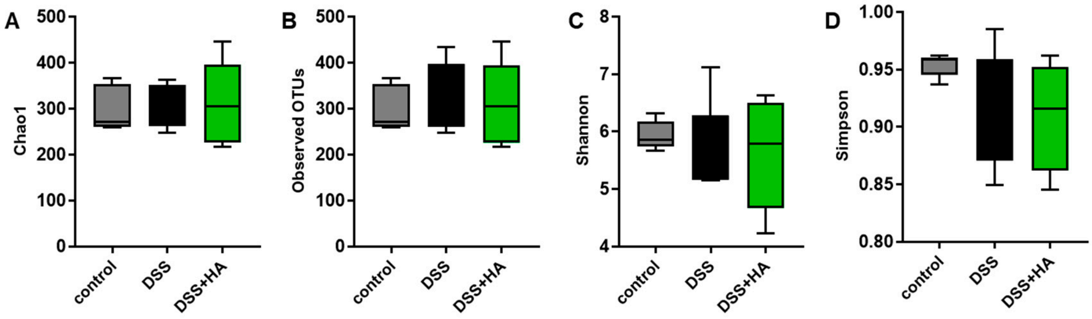

Figure S2. HA intervention demonstrates no significant influence on the α-diversity of GM. (**A−D**) The α-diversity analysis of the GM at the OTU level, determined by the Chao1, Observed OTUs, Shannon and Simpson index. Data are expressed as mean±SE, n = 4−5.

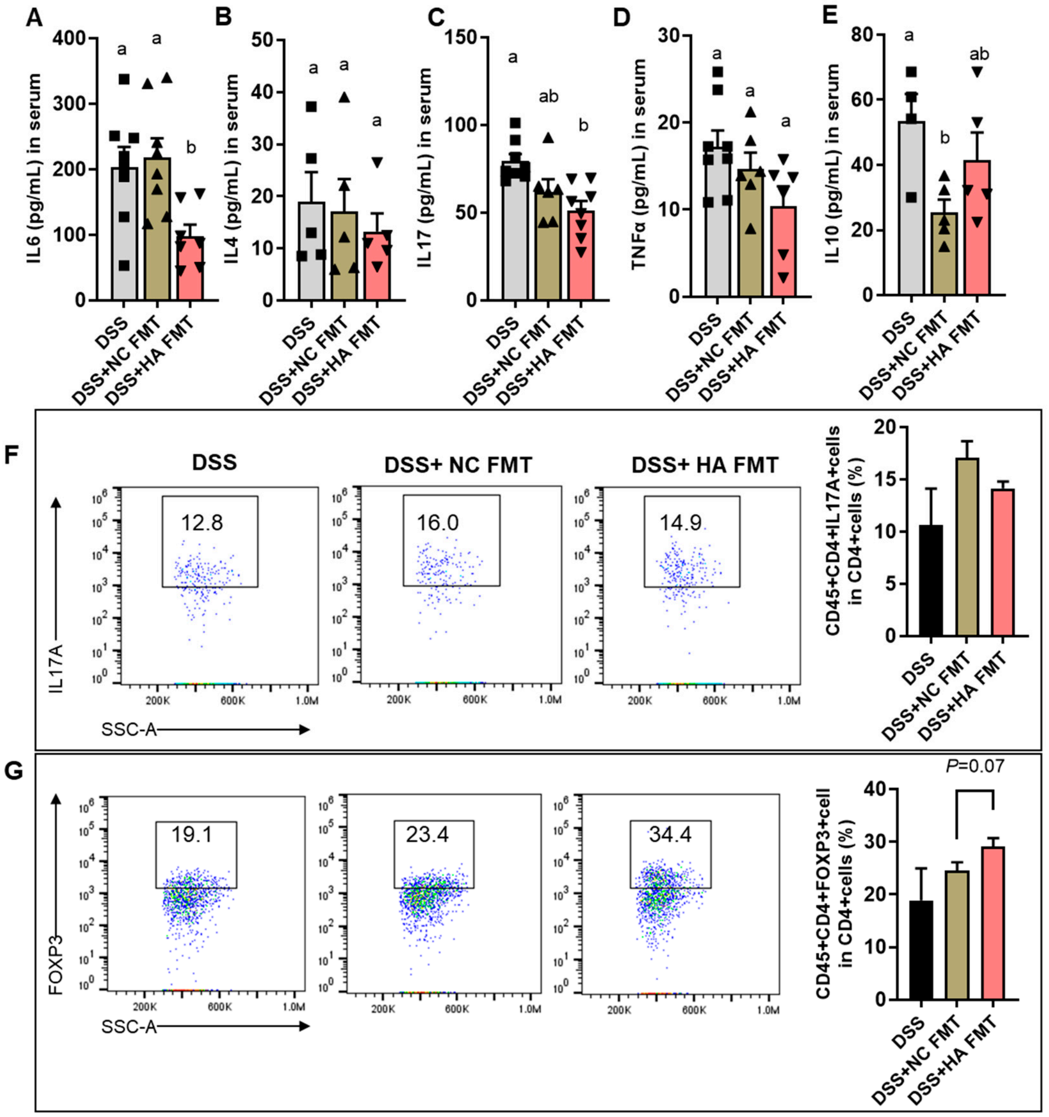

Figure S3. The effects of FMT from colitis mice treated with HA on inflammatory factors and immune cells. (**A−E**) Concentrations of IL4, IL6, TNFα, IL17 and IL10 in serum. (**F** and **G**) The proportion of Th17 (CD45+ CD4+ IL17A+) cells and Treg (CD45+ CD4+FOXP3+) cells. The color of the dots represents the frequency of cell occurrence. Redder represents greater cell density. Data are expressed as mean±SE, n = 4−6, and significant differences (P<0.05) are denoted by a, b.
